# Supplementary material for: Prenatal S-Adenosine Methionine (SAMe) Induces Changes in Gene Expression in the Brain of Newborn Mice That Are Prevented by Co-Administration of Valproic Acid (VPA)
Source: Int J Mol Sci. 2020 Apr 18;21(8):2834. doi: 10.3390/ijms21082834 (PMC7215397; doi:10.3390/ijms21082834)
Supplement: Supplementary file 1 [file ijms-21-02834-s001.pdf]

Supplementary figure 1: volcano plot SAMe versus control im males (A) and females (B).

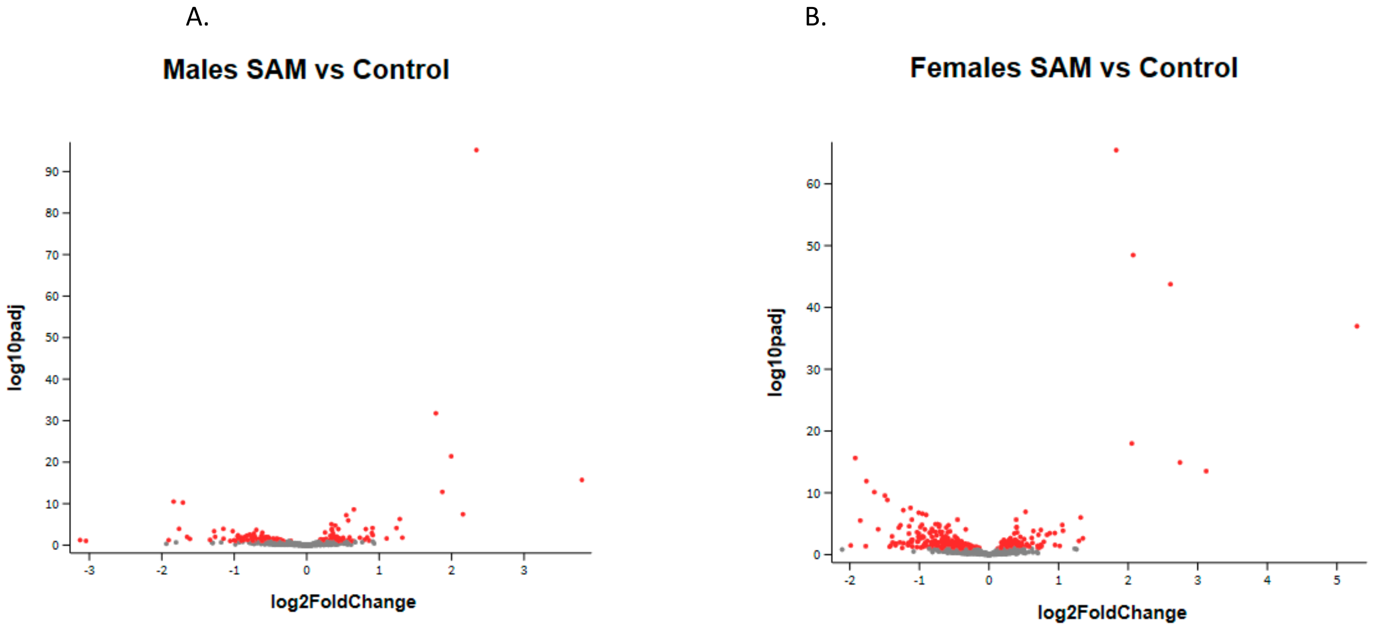

Supplementary figure 1. A,**B** are a volcano plot of log 2 fold-change (x-axis) versus −log 10 adjusted p-value (y-axis, representing the probability that the gene is differentially expressed). Every dot represents one gene. Red dots: genes with statistically significant change compared to controls. Grey dots: no statistical change.

Supplementary table 1: Full list of genes significantly changed in females after SAMe administration

symbol	log2 fold change	p value	ajd p value	% change control-sam
Vegfa	1.829	4.63E-69	3.57E-66	292%
Slc2a1	2.072	8.84E-52	3.40E-49	445%
Jun	2.608	6.80E-47	1.75E-44	449%
Fos	5.288	5.71E-40	1.10E-37	4294%
Flt1	2.053	6.72E-21	1.03E-18	272%
Thy1	-1.921	1.87E-18	2.40E-16	-66%
Egr1	2.745	1.11E-17	1.22E-15	664%
Npas4	3.121	3.18E-16	3.06E-14	479%
Gfap	-1.761	1.50E-14	1.29E-12	-59%
Fcrls	-1.647	9.75E-13	7.50E-11	-68%
Spi1	-1.496	4.01E-12	2.81E-10	-26%

Cx3cr1	-1.46	2.29E-11	1.47E-09	-66%
Htr1a	-1.128	4.77E-10	2.82E-08	-63%
C1qb	-1.231	1.19E-09	6.55E-08	-31%
Cdc40	0.5253	2.32E-09	1.19E-07	59%
Calm1	-1.009	3.50E-09	1.68E-07	-54%
C1qc	-0.9557	5.35E-09	2.42E-07	-24%
Stx1a	-0.9024	8.78E-09	3.76E-07	-54%
Icam1	1.318	2.42E-08	9.79E-07	415%
Cd68	-1.109	6.29E-08	0.0000022	-6%
Map2k2	-0.4522	6.10E-08	0.0000022	-26%
Polr2b	0.3907	5.95E-08	0.0000022	28%
Aqp4	-1.849	9.32E-08	3.119E-06	-61%
Gusb	-0.768	3.88E-07	0.00001194	-28%
Polr2l	-0.7367	3.78E-07	0.00001194	-43%
Cd44	-0.9637	5.08E-07	0.00001506	579%
Lypla1	-0.7132	5.48E-07	0.00001561	-3%
Nostrin	1.053	5.74E-07	0.0000158	91%
Mmp9	-1.272	6.58E-07	0.00001738	343%
Tada2b	-0.5816	6.77E-07	0.00001738	-42%
C1qa	-1.147	1.03E-06	0.00002557	-23%
Frmpd4	-0.7174	1.363E-06	0.0000328	-58%
Csf1r	-0.9855	1.571E-06	0.00003632	-22%
Gnai3	0.3987	1.604E-06	0.00003632	34%
Pink1	0.3945	1.762E-06	0.00003876	25%
Amph	-0.6141	1.89E-06	0.00004043	-48%
Cck	-1.295	2.079E-06	0.00004326	-57%
Gabra4	-0.8194	3.343E-06	0.00006773	-42%
Ccl12	-1.592	4.084E-06	0.00008063	12%
Gpd1l	-0.3336	4.414E-06	0.00008289	-28%
Nmb	-0.921	4.407E-06	0.00008289	-35%
Pecam1	0.7452	5.632E-06	0.0001033	171%
Grin1	-0.8108	7.923E-06	0.0001391	-42%
Tnfrsf12a	1.064	7.947E-06	0.0001391	372%
Atp7a	0.6386	8.925E-06	0.0001527	60%
Tgfb1	-0.5603	1.009E-05	0.0001689	166%
Fgf12	-0.7046	1.185E-05	0.0001941	-46%
Ap4s1	-0.676	0.0000134	0.0002109	-27%
Syt1	-0.7693	1.342E-05	0.0002109	-51%
Cycs	-1.035	1.547E-05	0.0002383	-54%
Lrrc4	-0.5941	1.616E-05	0.000244	-46%
Gtf2ird1	0.4049	1.785E-05	0.0002643	34%

Stambpl1	-0.8521	1.838E-05	0.0002671	-50%
Angpt2	0.9458	2.335E-05	0.000333	179%
Tbp	0.8721	2.517E-05	0.0003524	134%
Ltbr	-0.8446	2.581E-05	0.0003549	5%
Cntn1	-1.007	2.913E-05	0.0003935	-64%
Sgpl1	0.3638	2.974E-05	0.0003949	44%
Casp1	-1.15	3.089E-05	0.0004031	-46%
Cplx1	-0.6845	0.0000463	0.0005942	-53%
Insr	0.7119	5.021E-05	0.0006338	55%
Notch1	0.8328	5.137E-05	0.0006379	53%
Apoe	-0.7521	6.356E-05	0.0007768	-48%
Gabrg2	-0.6242	9.161E-05	0.001085	-45%
Psmb8	-0.9522	9.032E-05	0.001085	1%
Ppp3cc	-0.4895	9.504E-05	0.001109	-25%
Ache	-1.392	9.696E-05	0.001114	-41%
P2ry12	-1.015	0.0001143	0.001295	-58%
Grm5	-0.6223	0.0001212	0.001353	-50%
Sox9	0.4508	0.0001249	0.001374	30%
Cx3cl1	-0.8179	0.0001299	0.001409	-55%
Sncb	-0.9542	0.0001362	0.001457	-64%
Cyp4x1	-0.9367	0.0001494	0.001576	-61%
Pdgfrb	0.6144	0.0001888	0.001965	176%
Hras	-0.5886	0.0002007	0.00206	-25%
Lsm2	-0.4604	0.0002125	0.002153	-15%
Srsf4	0.3086	0.0002159	0.002159	5%
Ptgs2	1.35	0.0002374	0.002344	763%
Tgfbr2	-0.6628	0.0002478	0.002416	124%
Arhgap44	-0.6632	0.0002542	0.002446	-50%
Prkcq	-0.9832	0.0002645	0.002515	-41%
Msn	0.5056	0.0003312	0.00311	143%
Trem2	-1.099	0.0004228	0.003888	-30%
Xab2	0.3762	0.0004241	0.003888	35%
Ccr5	-1.112	0.0004676	0.00423	-65%
Sec23a	0.2121	0.0004725	0.00423	13%
Adra2a	-0.6786	0.0004922	0.004307	-51%
Cul2	-0.4459	0.0004978	0.004307	-24%
Fmr1	0.2912	0.0004932	0.004307	24%
Mta1	0.2889	0.0005121	0.004381	22%
Atp6v0e2	-0.632	0.0005577	0.004719	-28%
Ptdss2	-0.5305	0.0006046	0.00506	-16%
Taf4b	-0.776	0.0006296	0.005213	-3%

Cacnb4	-0.4683	0.0006433	0.00527	-25%
Gng2	0.3086	0.0006609	0.005357	-6%
Hpgds	-0.6112	0.0006906	0.005539	-27%
Cers6	0.3949	0.0007451	0.005915	2%
Ctse	1.292	0.0007861	0.006176	233%
Taz	0.3593	0.0007983	0.006209	50%
Grin2a	-0.8834	0.0008192	0.006308	-31%
Cul3	0.2309	0.0008342	0.006359	10%
Igf1	-1.024	0.0009308	0.007027	254%
Gal3st1	-1.106	0.0009769	0.007303	-48%
Rab3a	-0.3921	0.001047	0.007751	-35%
Cds1	-0.8079	0.001083	0.007943	-26%
Gnai1	-0.4167	0.0011	0.00799	-28%
Txnl1	-1.024	0.00112	0.00806	-55%
Nrg1	0.7881	0.001183	0.008436	7%
Glrb	-0.51	0.001214	0.008573	-37%
Epha7	-0.3922	0.001268	0.008875	-37%
Ntf3	-1.121	0.00132	0.009154	-26%
Adora1	-0.5725	0.001352	0.00921	-32%
Gnb5	-0.3937	0.001341	0.00921	-17%
Entpd4	-0.419	0.001375	0.009287	7%
Snap91	-0.6177	0.00139	0.009305	-50%
Mmp14	0.4014	0.001415	0.009365	199%
Taf10	-0.4497	0.001423	0.009365	-18%
Smpd4	0.3523	0.00149	0.009725	33%
Abl1	0.425	0.001539	0.009959	36%
P2rx4	-0.8301	0.001569	0.01007	-10%
Cul1	0.215	0.001625	0.01034	22%
Tlr4	-1.279	0.001682	0.01062	65%
Fyn	0.177	0.001725	0.0107	-6%
Gtf2h1	0.2783	0.001729	0.0107	16%
Wfs1	-0.7382	0.001737	0.0107	-34%
Vip	-1.385	0.001793	0.01095	-15%
Gabra1	-0.7208	0.001862	0.01129	-19%
Cadps	-0.6395	0.001919	0.01154	-49%
Prkca	-0.5427	0.001948	0.01163	-51%
Cd40	-0.9161	0.001979	0.01172	-18%
Gjb1	-1.155	0.002053	0.01207	81%
Nfkbia	0.4708	0.002089	0.01219	112%
Bnip3	0.6252	0.002193	0.0127	187%
Atf4	0.3649	0.002368	0.01361	55%

Camk4	-0.7809	0.002403	0.01364	-42%
Itgam	-0.7833	0.00241	0.01364	-23%
Bcas1	-1.227	0.002471	0.01389	-61%
Grm2	-0.6724	0.002513	0.01402	-48%
Tcerg1	0.4061	0.002629	0.01456	21%
Dnah1	-1.335	0.002942	0.01617	-64%
Stat1	-0.5995	0.00296	0.01617	-14%
Nptn	-0.3359	0.003184	0.01718	-23%
Scn2a1	-0.5504	0.003191	0.01718	-41%
Prkcb	-0.7577	0.003385	0.0181	-60%
Abat	-0.5048	0.00349	0.01853	-38%
Ptprr	-0.4682	0.00362	0.01909	-19%
Gpr37	-0.447	0.003697	0.01936	-18%
Map2k1	-0.4451	0.003721	0.01936	-1%
Rras	0.5434	0.003876	0.02003	357%
Grik2	0.4254	0.00422	0.02166	1%
Sf3b4	-0.5142	0.004378	0.02232	-41%
Akt1s1	-0.3491	0.004486	0.02257	-5%
Atp6v1d	-0.2915	0.004524	0.02257	-23%
Camk2b	-0.3021	0.004544	0.02257	-29%
Ube2n	-0.5176	0.004536	0.02257	-42%
Katna1	0.4364	0.004581	0.02261	84%
Trim28	0.2895	0.004757	0.02333	13%
Prkcg	-0.4571	0.004854	0.02366	-30%
Cacna1a	-0.9128	0.005074	0.02457	-29%
Dll4	0.5491	0.005342	0.02571	91%
Epha3	0.7445	0.005528	0.02644	85%
Ehmt1	0.3193	0.005689	0.02704	17%
Acaa1a	-0.9334	0.005915	0.02777	-24%
Gabrr1	-1.165	0.005886	0.02777	-54%
Axin2	-0.3996	0.006212	0.02786	-20%
Cldn15	0.9492	0.006049	0.02786	239%
Cldn5	-0.4066	0.006113	0.02786	-24%
Fasl	-1.158	0.006185	0.02786	-56%
Myc	-0.3796	0.006004	0.02786	-26%
Raf1	0.247	0.006136	0.02786	17%
Sf3b2	0.1885	0.006007	0.02786	11%
Snca	-0.4503	0.006224	0.02786	-39%
Xbp1	-0.5163	0.006541	0.02912	2%
Gnptab	-0.3979	0.006765	0.02994	-32%
Smyd1	-0.9203	0.006829	0.03005	877%

Ide	-0.4288	0.006946	0.03039	-21%
Ran	-0.4567	0.007052	0.03068	-33%
Atp6v0c	-0.4463	0.007095	0.03069	-38%
S100b	-1.341	0.007263	0.03107	-4%
Sox10	-0.7491	0.007237	0.03107	-42%
Nefh	-1.987	0.007716	0.03282	-67%
Erbb3	-1.41	0.007818	0.03308	372%
Kras	-0.6103	0.008084	0.03401	-39%
Dgke	-0.2538	0.008213	0.03437	-31%
Naglu	-0.3859	0.008352	0.03476	20%
Igf1r	0.7006	0.009022	0.03657	90%
Mmp24	-0.27	0.008889	0.03657	-21%
Mta2	0.383	0.008975	0.03657	87%
Nsf	-0.6012	0.009023	0.03657	-49%
Rdx	0.2224	0.00896	0.03657	22%
Aif1	-0.9421	0.009146	0.03687	-67%
Htr5a	-0.838	0.009234	0.03696	-50%
Pmp22	0.4385	0.009264	0.03696	400%
Acvrl1	0.5481	0.009484	0.03764	158%
Hdac2	0.2063	0.009844	0.03887	4%
Stab1	-0.6414	0.01014	0.03984	-14%
Napsa	1.016	0.01028	0.04017	709%
Atp6v1e1	-0.418	0.01062	0.04129	-17%
Crtc2	0.4835	0.01073	0.04131	95%
Slc11a1	-0.7996	0.01069	0.04131	-47%
Prkaca	-0.3252	0.01092	0.04182	24%
Cnr1	0.4386	0.01099	0.04189	19%
Cxcr4	0.7549	0.01127	0.04276	64%
Slc6a4	-1.771	0.01157	0.04368	-81%
Nes	0.5764	0.01208	0.04539	45%
Nefl	-1.101	0.01293	0.04832	-63%
Atp6v1g2	-0.6445	0.01309	0.04869	-38%
Jam3	0.3124	0.01353	0.05008	84%
Ptprn2	-0.4203	0.01371	0.05052	-45%
Gabrp	-1.427	0.01441	0.05283	82%
Arrb2	-0.2636	0.01469	0.05337	-23%
Dgkb	-0.7163	0.01465	0.05337	-53%
Atxn3	0.3056	0.01488	0.05379	24%
Creb1	0.3545	0.01496	0.05384	25%
Ikbkb	-0.2364	0.01507	0.05398	-4%
Keap1	-0.1999	0.01533	0.05464	6%

Plxnb3	-0.9453	0.01549	0.05497	11%
Grm8	-0.8077	0.0157	0.05533	-61%
Lsr	-0.4244	0.01585	0.05533	-15%
Pgk1	0.3477	0.01574	0.05533	80%
Plcl2	-0.1752	0.01588	0.05533	-23%
Atp2b3	-0.4976	0.01668	0.05759	-23%
Rit2	-0.8075	0.01668	0.05759	-58%
Hexb	-0.374	0.01706	0.05863	-3%
Bax	-0.3435	0.01737	0.05939	-24%
Src	0.3063	0.01743	0.05939	-5%
Nme5	-0.5168	0.01841	0.06246	-32%
Olfm3	-0.8464	0.01868	0.06308	-45%
Cntn4	-0.8727	0.01901	0.06363	-51%
Park7	-0.3311	0.01897	0.06363	-10%
Th	-1.035	0.01922	0.06408	-50%
Mfn2	-0.3457	0.01945	0.06439	-25%
Sart1	-0.5298	0.01948	0.06439	-26%
Bche	-0.6921	0.01971	0.06458	48%
Xk	-0.5171	0.01967	0.06458	-37%
Fam126a	0.3263	0.02041	0.06658	24%
Tnfrsf10b	0.7323	0.02052	0.06665	409%
Pcsk2	-0.5121	0.02104	0.06808	-52%
Il4ra	-0.5678	0.0214	0.06896	10%
Actn1	0.2733	0.02172	0.06969	52%
Cers4	0.2183	0.02185	0.0698	-2%
Man2b1	-0.2775	0.02195	0.06985	14%
Il10ra	-0.7887	0.02207	0.06992	32%
Plcb2	-0.9781	0.02296	0.07247	3%
Irf8	-0.5999	0.02342	0.07359	-16%
Map2	-0.2237	0.02408	0.07536	-31%
Hmox1	-0.5024	0.02423	0.07555	46%
Lars	0.1721	0.02505	0.07776	14%
Casp8	-0.4877	0.02518	0.07787	86%
Sf3a2	0.1809	0.02578	0.07941	6%
Pkn1	0.3001	0.02602	0.07952	19%
Slc4a10	-0.5633	0.02602	0.07952	-30%
L1cam	-0.4306	0.02652	0.08072	-48%
Chd4	0.3946	0.02666	0.08081	21%
Becn1	-0.1384	0.02694	0.08136	-17%
Cntnap1	-1.246	0.02708	0.08146	-11%
Cab39	0.1596	0.02772	0.08305	9%

Asb7	-0.2693	0.02798	0.08351	-12%
Arc	0.7039	0.02842	0.08418	64%
Egfl7	-0.3692	0.02835	0.08418	24%
Dcx	0.3434	0.02874	0.0848	-2%
Slc32a1	-0.3877	0.02949	0.08668	-46%
Lamp1	-0.221	0.02967	0.08686	-1%
Scamp2	-0.2118	0.02986	0.08709	-1%
Bdnf	-0.7902	0.03115	0.09051	-25%
Car2	-0.4664	0.03137	0.0908	-21%
Homer1	0.3016	0.03192	0.09205	2%
Kcna1	-0.5278	0.03236	0.09275	115%
Ppp2r5c	-0.2141	0.03252	0.09275	-16%
Slu7	0.2387	0.03244	0.09275	16%
Eif2s1	-0.2926	0.03278	0.09309	-1%
Pten	-0.2519	0.03288	0.09309	-20%
Tnfrsf1b	-0.6754	0.03333	0.094	-30%
Islr2	0.4042	0.03376	0.09487	29%
Ap2a2	-0.2503	0.03588	0.09855	-8%
B4galt6	-0.3506	0.0358	0.09855	-41%
Cdk2	0.3102	0.03529	0.09855	179%
Cxcl16	-0.5964	0.03604	0.09855	62%
Mapk3	-0.2617	0.03538	0.09855	-2%
Ngfr	-0.6918	0.0359	0.09855	45%
Rac1	0.1298	0.03584	0.09855	5%
Unc13a	-0.4095	0.03609	0.09855	-46%

Supplementary table 2: Full list of genes significantly changed in males after SAMe administration

symbol	log2 fold change	p value	ajd p value	% change control-SAMe
Vegfa	2.343	8.60E-99	6.62E-96	390%
Slc2a1	1.783	4.55E-35	1.75E-32	263%
Jun	1.994	1.52E-24	3.90E-22	317%
Fos	3.798	1.01E-18	1.95E-16	1516%
Flt1	1.872	9.79E-16	1.51E-13	219%
Gfap	-1.837	2.50E-13	3.21E-11	-74%
Thy1	-1.709	4.97E-13	5.47E-11	-68%
Gng2	0.6513	2.62E-11	2.52E-09	43%

Egr1	2.157	4.28E-10	3.66E-08	375%
Pink1	0.5459	7.74E-10	5.96E-08	49%
Nostrin	1.285	7.34E-09	5.14E-07	138%
Gtf2ird1	0.5731	1.75E-08	0.000001121	36%
Sec23a	0.3418	1.49E-07	0.000008821	27%
Polr2b	0.3943	3.48E-07	0.00001913	23%
Insr	0.909	0.0000016	0.00007701	49%
Tnfrsf12a	1.239	1.562E-06	0.00007701	218%
Aqp4	-1.762	2.584E-06	0.0001157	-69%
Fcrls	-1.15	2.705E-06	0.0001157	-52%
Cul3	0.3468	3.06E-06	0.000124	24%
Cdc40	0.4373	3.528E-06	0.0001348	32%
Pecam1	0.8162	3.675E-06	0.0001348	52%
Lypla1	-0.6954	5.885E-06	0.000206	-30%
Cab39	0.3489	7.391E-06	0.0002474	25%
Cx3cr1	-1.021	1.326E-05	0.0004254	-51%
Cck	-1.278	1.428E-05	0.00044	-57%
Fyn	0.2538	2.937E-05	0.0008699	15%
Lrrc4	-0.6127	3.367E-05	0.0009603	-37%
Bnip3	0.8995	4.418E-05	0.001215	108%
Mta1	0.3626	5.241E-05	0.001391	27%
Cplx1	-0.7283	6.242E-05	0.001602	-40%
Apoe	-0.7861	0.0001092	0.002713	-40%
Angpt2	0.9119	0.0001602	0.003855	83%
Taf4b	-0.9481	0.000175	0.004084	-59%
Cd68	-0.825	0.0002223	0.005034	-48%
Glrb	-0.6242	0.0002465	0.005423	-30%
Gnai3	0.326	0.0002759	0.0059	22%
Rab2a	0.3445	0.0003068	0.006385	22%
Cers4	0.3672	0.0003313	0.006712	13%
C1qc	-0.6155	0.0004158	0.008146	-28%
Taz	0.4091	0.0004232	0.008146	26%
Stab1	-0.9542	0.0004567	0.008578	-54%
Cd44	-0.7152	0.0005058	0.009272	-40%
Csf1r	-0.7651	0.000518	0.009276	-40%
C1qb	-0.7459	0.0005787	0.01012	-37%
Ccl12	-1.264	0.0005911	0.01012	-68%
C1qa	-0.8551	0.0006604	0.01046	-40%
Calm1	-0.629	0.0006551	0.01046	-31%
Gtf2a1	0.4187	0.0006642	0.01046	29%
Nmb	-0.7414	0.0006732	0.01046	-43%

Ptdss2	-0.5637	0.0006927	0.01046	-27%
Vip	-1.651	0.0006884	0.01046	-81%
Cadm3	0.5912	0.0009726	0.01337	37%
Cldn5	-0.5303	0.0009361	0.01337	-28%
Frmpd4	-0.5357	0.0009416	0.01337	-44%
Icam1	0.8363	0.0009436	0.01337	110%
Mmp9	-0.9047	0.0009723	0.01337	-55%
Spi1	-0.7208	0.001045	0.01412	-40%
Atp7a	0.5027	0.001199	0.01565	25%
Ctse	1.32	0.001192	0.01565	226%
Acvrl1	0.7338	0.001239	0.0159	45%
Gusb	-0.5271	0.001338	0.01689	-31%
Sox9	0.4038	0.001379	0.01712	30%
Tgfbr2	-0.625	0.001523	0.01862	-41%
Tcerg1	0.4526	0.001904	0.02291	21%
Cldn15	1.103	0.002108	0.02437	213%
Grin1	-0.6005	0.002107	0.02437	-36%
Lsm2	-0.4112	0.00212	0.02437	-19%
Ntf3	-1.148	0.002434	0.02731	-55%
Polr2l	-0.4695	0.002447	0.02731	-23%
Abl1	0.4381	0.002595	0.02854	11%
Adora1	-0.5798	0.002643	0.02866	-37%
Srsf4	0.2682	0.002808	0.03003	5%
Taf10	-0.4534	0.002892	0.03051	-20%
Casp8	-0.7193	0.002973	0.03094	-44%
S100b	-1.612	0.003057	0.03139	-71%
Dcx	0.4928	0.003667	0.03716	29%
Ap4s1	-0.4819	0.003997	0.03899	-24%
Car2	-0.6711	0.004022	0.03899	-32%
Comt	-0.4637	0.004102	0.03899	-18%
Rac1	0.1923	0.003915	0.03899	10%
Sf3b4	-0.5562	0.004055	0.03899	-39%
Akt1s1	-0.3772	0.004433	0.04112	-19%
Casp1	-0.8956	0.004385	0.04112	-66%
Tnfrsf11b	-0.7499	0.004551	0.04171	-43%
Htra2	-0.4212	0.005085	0.04568	-22%
Neo1	0.3943	0.005102	0.04568	18%
Ipcef1	-0.635	0.005323	0.04649	-35%
Lars	0.2297	0.005374	0.04649	12%
Pik3ca	0.4425	0.005278	0.04649	23%
Eif2s1	-0.4075	0.005854	0.04936	-17%

Igf1r	0.7978	0.005905	0.04936	40%
Prkcg	-0.4826	0.005827	0.04936	-38%
Slu7	0.3305	0.005962	0.04936	14%
Htr1a	-0.5245	0.006193	0.05073	-29%
Tlr4	-1.335	0.006347	0.05144	-87%
Prkca	-0.5152	0.00646	0.05181	-38%
Sox10	-0.8174	0.006533	0.05186	-51%
Man2b1	-0.3536	0.006649	0.05224	-20%
Akt3	0.3459	0.007103	0.05524	21%
Pllp	-0.8722	0.007348	0.05658	-59%
Ccl5	-3.128	0.007802	0.05948	-11%
Nlrp3	-1.905	0.008	0.06039	-88%
Hif1a	0.1814	0.008848	0.06615	13%
Cul1	0.1915	0.009024	0.06681	15%
Ache	-1.002	0.009216	0.06683	-50%
Bcl2l1	0.2217	0.009291	0.06683	17%
Cers6	0.3276	0.009461	0.06683	15%
Gnaq	0.401	0.009411	0.06683	19%
Gucy1b3	-0.4391	0.009408	0.06683	-26%
Axin2	-0.407	0.009642	0.06749	-30%
Acaa1a	-0.9397	0.01038	0.07201	-42%
Notch1	0.5677	0.01059	0.07282	19%
Hdac2	0.2194	0.01085	0.07391	17%
P2ry12	-0.71	0.01115	0.07533	-37%
L1cam	-0.5309	0.01136	0.07605	-39%
Ngf	-0.9027	0.01202	0.07854	-59%
Raf1	0.2443	0.01189	0.07854	20%
Trpm2	-0.7433	0.01204	0.07854	-51%
Amph	-0.3448	0.01294	0.0824	-27%
Galc	0.2853	0.01295	0.0824	22%
Rras	0.4986	0.01276	0.0824	52%
Ager	0.8604	0.01315	0.08299	69%
Hras	-0.421	0.01374	0.086	-16%
Cacnb4	-0.3554	0.01576	0.09057	-25%
Ccr5	-0.7892	0.01573	0.09057	-54%
Dll4	0.5237	0.01575	0.09057	33%
Drd1	-0.9762	0.01523	0.09057	-53%
Lrrc25	-0.7165	0.01573	0.09057	-52%
Ltbr	-0.5252	0.01575	0.09057	-27%
Map2k2	-0.2192	0.01461	0.09057	-12%
Pdpk1	0.2946	0.01504	0.09057	24%

Stx1a	-0.4115	0.01501	0.09057	-23%
Tgfb1	-0.3268	0.01527	0.09057	-21%
Wfs1	-0.6154	0.01525	0.09057	-41%
Sp100	-3.047	0.01633	0.09312	-52%
Itga7	-0.7745	0.01718	0.09629	-45%
Phf21a	0.4417	0.01744	0.09629	26%
Pla2g4b	0.6307	0.01751	0.09629	22%
Rdx	0.2187	0.01731	0.09629	12%
Src	0.3306	0.01742	0.09629	14%
Lpo	-1.057	0.01789	0.09703	-74%
Usp30	0.2179	0.01786	0.09703	6%
Gstp1	-0.647	0.01834	0.09878	-34%
